# An Augmented Reality Mobile App (Easypod AR) as a Complementary Tool in the Nurse-Led Integrated Support of Patients Receiving Recombinant Human Growth Hormone: Usability and Validation Study

**DOI:** 10.2196/44355

**Published:** 2023-04-21

**Authors:** Rosa Maria Baños, Laura-Maria Peltonen, Blaine Martin, Ekaterina Koledova

**Affiliations:** 1 Department of Personality, Evaluation and Psychological Treatment Faculty of Psychology University of Valencia Valencia Spain; 2 Centro De Investigación Biomédica en Red of Physiopathology of Obesity and Nutrition Carlos III Health Institute Madrid Spain; 3 Department of Nursing Science University of Turku Turku Finland; 4 Global Digital Health Ares Trading SA, an affiliate of Merck KGaA (Darmstadt, Germany) Eysins Switzerland; 5 Global Medical Affairs Cardiometabolic & Endocrinology The health care business of Merck KGaA Darmstadt Germany

**Keywords:** augmented reality, growth hormone, growth hormone deficiency, mobile app, mobile health, nurse, patient support program, telehealth, telemedicine, treatment

## Abstract

**Background:**

Children with growth hormone deficiency face the prospect of long-term recombinant human growth hormone (r-hGH) treatment requiring daily injections. Adherence to treatment is important, especially at treatment initiation, to achieve positive health outcomes. Historically, telenursing services embedded in patient support programs (PSPs) have been a valid approach to support r-hGH treatment initiation and patient education and facilitate adherence by identifying and optimizing appropriate injection techniques. The development of mobile phones with augmented reality (AR) capabilities offers nurses new tools to support patient education.

**Objective:**

To investigate experiences among nurses of a new mobile phone app developed to support patient training with a phone-based PSP for r-hGH treatment.

**Methods:**

In 2020, the Easypod AR mobile app was launched to support nurse-driven telehealth education for patients initiating r-hGH therapy with the Easypod electromechanical auto-injector device. Nurses who were part of PSPs in countries where the Easypod AR app had been launched or where training was provided as part of an anticipated future launch of the app were invited to participate in an online survey based on the Mobile App Rating Scale to capture their feedback after using the app.

**Results:**

In total, 23 nurses completed the online questionnaire. They positively rated the quality of the app across multiple dimensions. The highest mean scores were 4.0 for engagement (ie, adaptation to the target group; SD 0.74), 4.1 (SD 0.79) for functionality (navigation) and 4.1 (SD 0.67) for aesthetics (graphics). Responses indicated the potential positive impact of such a tool on enhancing patient education, patient support, and communication between patients and PSP nurses. Some participants also suggested enhancements to the app, including gamification techniques that they felt have the potential to support the formation of positive treatment behaviors and habits.

**Conclusions:**

This study highlights the potential for new digital health solutions to reinforce PSP nurse services, including patient education. Future studies could explore possible correlations between any behavioral and clinical benefits that patients may derive from the use of such apps and how they may contribute to support improved patient experiences and treatment outcomes.

## Introduction

Growth hormone deficiency (GHD), in which the pituitary gland fails to produce a sufficient amount of growth hormone (GH) during childhood, affects between 1 in 3500 and 1 in 4000 children in the United Kingdom [1]. It exposes children to the prospect of long-term recombinant human GH (r-hGH) treatment that requires daily injections over several years [2]. This can lead to challenges with adherence to therapy [3] and often puts a substantial social and emotional burden on patients and their families, affecting both well-being and quality of life [4]. Furthermore, suboptimal adherence to therapy is inevitably also associated with less favorable clinical outcomes and higher health care costs [2].

Successfully achieving full height potential with r-hGH therapy depends on maintenance of optimal dosing schedules, adherence to therapy, and early treatment initiation [2,5]. Indeed, a systematic review by Graham et al [6] reported that up to 71% of children with GHD and their families were nonadherent to their r-hGH therapy as prescribed, with key modifiable factors including a lack of knowledge and understanding of their condition and treatment, discomfort and pain associated with injections, and the quality of the health care professional (HCP)-patient relationship. Treatment adherence is one of the main drivers of health outcomes [7,8], and this is especially important at treatment initiation, when needle anxiety might be more prevalent [9] and sufficient competence is needed to ensure proper injection techniques are followed.

The need for frequent injections over a long period of time in the treatment of GHD has driven the development of medical devices that facilitate the subcutaneous delivery of medication and thus have the potential to improve patients’ adherence to therapy. In recent years, several devices have been developed for the administration of GH. So far, different types of GH injection devices have been introduced, such as needle syringes, injection pens, self-injection pens, and electronic devices. Despite advances in connected injection delivery devices and digital health, there is still room for improvement in the management of GH treatment. Studies carried out with patients, caregivers, doctors, and nurses experienced in the administration of GH indicate that an optimal device for the administration of GH should have the following characteristics: (1) reliability, (2) ease of use, (3) effective control via a good monitoring system, (4) absence of pain during injection; (5) safety in use and storage, and (6) a minimum number of steps before injection preparation [10,11]. In addition to the evolution of delivery devices, a wide range of digital solutions have been developed to support GH treatment [12-14].

Such medical devices require patient education, which is often delivered via patient support programs (PSPs) [15,16]. These are structured programs that offer services through patient or patient-caregiver interactions, and given the focus on patient education and adherence support, PSPs are often delivered by nurses. They are predominantly designed to support the education of patients who must follow complex treatment modalities that require the use of technology, and they may also be part of pharmacovigilance responsibilities under medical device regulations [17]. PSPs have been shown to help patients and their families from treatment initiation through the maintenance and transition period to adult care, if required [15], and they are often provided by manufacturers of specific drugs or medical devices according to the legislation of each country.

For many years, telenursing services embedded in PSPs have been a valid approach to support treatment initiation. Nurses involved in PSPs provide patient education to facilitate adherence by identifying and optimizing appropriate injection techniques. The emergence of more advanced mobile phone technology with augmented reality (AR) capabilities offers nurses new tools to support patient education.

Outside the GH therapy area, emerging research is showing the potential of using AR to complement pediatric patient education in areas such as diabetes [18], asthma [19], and chronic pain [20]. Also, a recent review of the use of AR in nurse training revealed that experiences of using such technology were positive overall [21]; however, the included studies mostly focused on AR applications in nurse education and did not address patient education. Overall, there is a lack of knowledge on how nurses involved in patient education perceive AR and other similar digital solutions and their potential to deliver effective patient education.

The evaluation of digital solutions and mobile apps to support patient education and self-management has been found to be a complex multidimensional process addressing different aspects, such as usability and engagement, that can be framed across multiple theoretical frameworks. To address the challenge of the quality evaluation of mobile health solutions and to evaluate the extent to which they are suitable for the target population, Stoyanov et al [22] created a new validated survey, the result of a systematic evaluation of previous research—the Mobile App Rating Scale (MARS).

Given the need for improvements in r-hGH treatment, this study aims to analyze emerging digital solutions and the opinions of nurses concerning the use of tools such as the Easypod AR mobile app to educate and support patients receiving GH treatment for growth disorders as part of a PSP.

## Methods

### Study Design

The presented study is a cross-sectional survey with a single evaluation time. Study participants comprised PSP nurses and HCPs who support patients and their families during the initiation of r-hGH treatment administered with the connected Easypod device.

### Participants

All participating PSP nurses worked as third-party providers who were managed by the respective local affiliates of the health care business of Merck KGaA (Darmstadt, Germany) and offered the Easypod AR mobile app to patients as part of their r-hGH treatment initiation and education. They were invited by email by EK to respond to an online survey. There was no other involvement or influence by local affiliates, and each PSP nurse was asked to provide their individual consent to the survey and to respond independently. The invited nurses were from countries where the Easypod AR mobile app was already integrated into the PSP nurse care routine, including Hong Kong, Taiwan, Germany, Singapore, the United Kingdom, Australia, and South Korea. PSP nurses in Argentina also participated, based on preparation and training they received when the app was launched there in 2022.

### Data Collection

Participants anonymously accessed an online form where they evaluated and commented on their experience with the use of the Easypod AR mobile app in their work educating patients in receiving r-hGH treatment. The researchers did not collect the participants’ contact details, as EK distributed the invitation to take part in the study to all eligible participants. The online questionnaire was based on the validated MARS questionnaire, which assesses, mostly using Likert scales (eg, 1=strongly disagree, 2=disagree, 3=neither agree nor disagree, 4=agree, and 5=strongly agree) [23], whether an intervention is perceived as entertaining, interesting, customizable, and interactive [22]. It also addresses usability and perceived impact in terms of self-management. Regarding the *association* dimension (the self-management behaviors that the app is aiming to impact), and following MARS guidelines, we adapted the questionnaire for the administration of r-hGH using the connected Easypod device (Multimedia Appendix 1 provides more details on the adaptation of this dimension). We did not use the MARS *information* dimension since the quality of the information within the app was out of the scope of the study. Furthermore, we added a few additional questions on the subjective quality of the mobile app in the context of using it for patient support (eg, the impact of COVID-19 on using the app to support patient education). Finally, the survey included a qualitative feedback element to acquire additional context and understanding of the mobile app using questions such as “Please enter any feedback suggestions for the improvement of Easypod AR” and “Please enter any positive feedback about your experience with Easypod AR.”

### Data Analysis

The data were analyzed using simple descriptive statistics, and the qualitative feedback received is presented together with these data.

### Ethics

This project follows the ethical and deontological principles marked by the principles of the Declaration of Helsinki and the Convention of the Council of Europe for the protection of human rights and the dignity of the human being regarding the applications of biology and medicine of Oviedo. Special care was taken with respect to informed consent of the PSP nurses, their voluntary participation, and their right to leave the study at any time. The project was approved by the Ethical Review Committee of the University of Valencia, Spain (1807083).

### Saizen Digital Health Ecosystem

**Figure 1:**
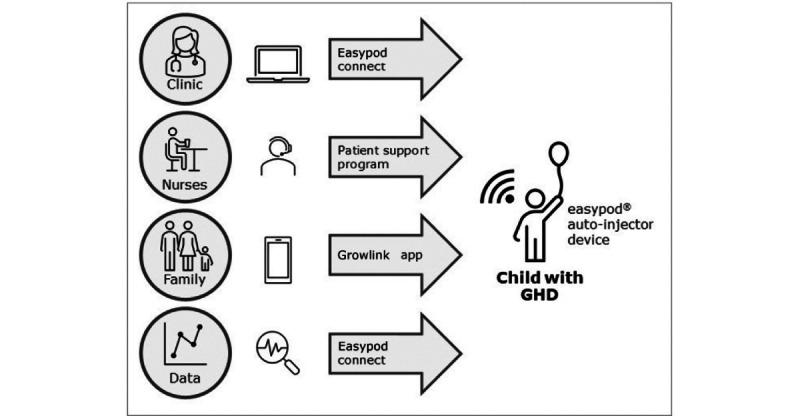
The Saizen digital health ecosystem. GHD: growth hormone deficiency.

This study took place within the context of treatment with r-hGH (somatropin; Saizen, the health care business of Merck KGaA, Darmstadt, Germany) and the r-hGH digital health ecosystem, comprising the Easypod connected injection device, Easypod Connect for HCPs, the Growlink app for patients and caregivers, the integrated PSP nurses, and the TuiTek adherence support behavior coaching program. This evolving platform of digital health solutions connects HCPs with their patients and their caregivers to support optimal treatment adherence and deliver HCP insights to support clinical decision-making to achieve the best possible growth outcomes. Figure 1 illustrates how patients using the connected Easypod device work with PSP nurses within the context of the r-hGH digital health system.

### Description of PSP

The participants in this study were r-hGH PSP nurses who worked for the services provided by the health care business of Merck KGaA, Darmstadt, Germany. These nurses provide support and training to patients receiving r-hGH. In addition, the prescribing HCPs had access to a remote dashboard to monitor patient adherence [24] to the Easypod device and facilitate communication between themselves and the patient and their caregiver [9]. Previous studies have shown high levels of adherence in patients using Easypod and also observed clinically significant changes in height and mean growth velocity [25].

### Easypod AR App

**Figure 2:**
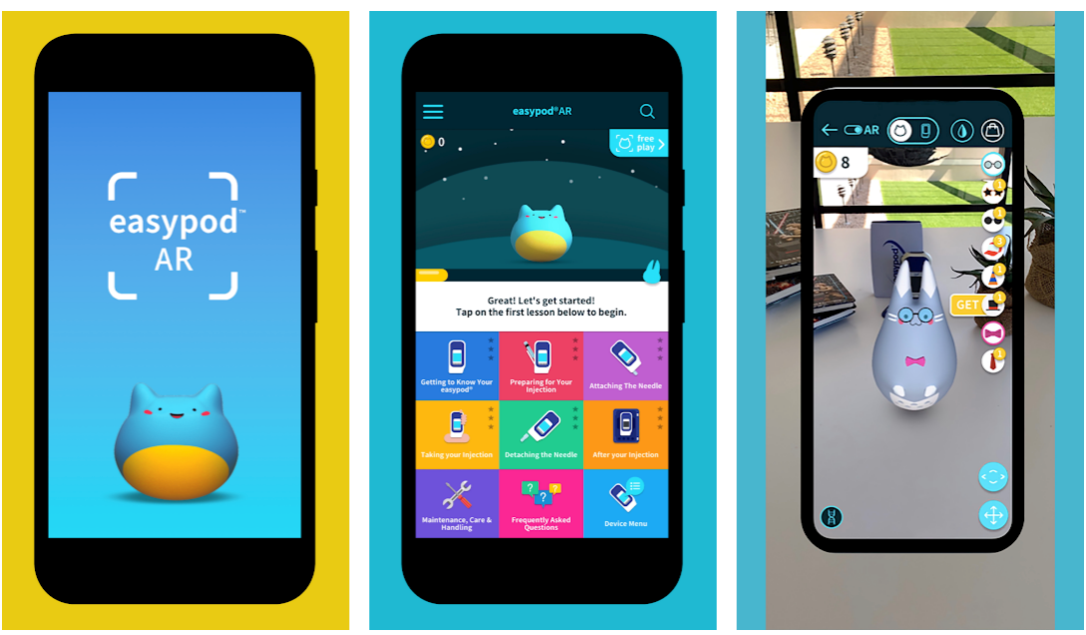
Screenshots of the Easypod AR mobile app.

The Easypod AR mobile app was specifically developed to help educate and support patients and their families using the connected Easypod device to administer r-hGH. It is a digital and interactive tool that contains the instructions for using the Easypod device to administer r-hGH (Figure 2). The tool aims to help patients better understand the steps of the injection process using AR technology to provide an immersive learning experience. The app is also intended to help HCPs resolve questions that patients or caregivers may have and provide support between visits.

### Setting

The Easypod AR app was launched in 2020 and had over 1000 users as of December 2021 in the countries where the study was performed (Hong Kong, Taiwan, Germany, Singapore, the United Kingdom, Australia, and South Korea). The average amount of time that these users spent with the app per session (defined as one user opening the mobile app) varied by country but averaged between 8 and 14 minutes, which is a considerable duration for a mobile app [26] and may reflect patient engagement during treatment initiation and PSP-delivered training.

## Results

In total, 23 nurses (of 24 to whom the email invitation was sent, ie, the total number of nurses who were part of PSPs in countries where the Easypod AR app had been launched or where training was provided as part of an anticipated future launch of the app) responded to the email invitation to participate in the study and completed the online questionnaire anonymously (Multimedia Appendix 2).

### Engagement, Functionality, and Aesthetics

The nurses reported average positive results in all 5 subdimensions of *engagement* (*entertainment*, *interest*, *customization*, *interactivity*, and *adaptation* to the target group). All reported mean scores were ≥3.0 (Table 1; Multimedia Appendix 1 provides details of the specific rating scales for each dimension). The highest mean score reported for *engagement* (adaptation to the target group) was 4.0 (SD 0.74). The lowest score (3.3, SD 0.97) was reported for *entertainment*, which aligns with qualitative feedback that suggested adding more engagement features such as gamification elements.

Within the dimension *functionality*, the highest score (4.1, SD 0.79) was reported for *navigation*, while the lowest (3.8, SD 1.03) was reported for *performance*. The qualitative feedback provided by the nurses showed that in some devices, the app was slower than desired, which might reflect the use of mobile devices with fewer technical features; this might have compromised the performance of the app, which has substantial 3D visualizations and AR.

Within the dimension related to *aesthetics*, the average score was positive for all elements, but the highest score was reported for *graphics* (4.1, SD 0.67) and the lowest score reported for *visual appeal* (3.7, SD 0.81).

**Table 1:** Scores per dimension from nurse participants (n=23). Multimedia Appendix 1 provides details of the specific rating scales for each dimension.

Dimensions/subdimensions		Scores, mean (SD)	Range
**Engagement**			
	Entertainment	3.3 (0.97)	2-5
	Interest	3.6 (0.72)	2-5
	Customization	3.5 (0.85)	2-5
	Interactivity	3.3 (1.10)	1-5
	Target group	4.0 (0.74)	3-5
**Functionality**			
	Performance	3.8 (1.03)	2-5
	Ease of use	3.9 (0.73)	3-5
	Navigation	4.1 (0.79)	3-5
	Gestural design	4.0 (0.77)	2-5
**Aesthetics**			
	Layout	4.0 (0.77)	2-5
	Graphics	4.1 (0.67)	3-5
	Visual appeal	3.7 (0.81)	2-5

### Association With Self-management

The MARS questionnaire also includes a *perceived association* dimension, which in this case was related to the administration of r-hGH using the connected Easypod device. The perceived impact scaled positively across all subdimensions, notably exceeding in *knowledge*, *emotions*, and *help-seeking* (Table 2).

**Table 2:** Perceived impact of Easypod AR by nurses in relation to administration of r-hGH via Easypod (n=23).

	Respondents, n (%)		
	Disagree or strongly disagree	Neutral	Agree or strongly agree
Emotions^a^	2 (9)	6 (26)	15 (65)
Behavior change^b^	1 (4)	8 (35)	14 (61)
Help-seeking^c^	1 (4)	7 (30)	15 (65)
Intention to change^d^	2 (9)	8 (35)	13 (57)
Attitudes^e^	1 (4)	9 (39)	13 (57)
Knowledge^f^	0 (0)	7 (30)	16 (70)
Awareness^g^	0 (0)	8 (35)	15 (65)

^a^Emotions: “The use of Easypod AR is likely to decrease the fear and anxiety associated with using this treatment procedure.”

^b^Behavior change: “The use of Easypod AR is likely to increase the proper and safe administration of this treatment procedure.”

^c^Help-seeking: “The use of Easypod AR is likely to encourage further help-seeking for correct treatment procedure via the Patient Support Program.”

^d^Intention to change: “The use of Easypod AR is likely to increase intentions/motivation to follow the correct treatment procedure.”

^e^Attitudes: “The use of Easypod AR is likely to change attitudes toward using the device properly and in a safe way.”

^f^Knowledge: “The use of Easypod AR app is likely to increase knowledge/understanding of using the correct treatment procedure properly and safely.”

^g^Awareness: “The use of Easypod AR app is likely to increase awareness of the importance of addressing the treatment procedure properly and safely.”

### Subjective Quality of Easypod AR

The subjective quality of the mobile app was high, with 70% (16 of 23) of the respondents saying that they would recommend it (Table 3). Also, the average rating was quite positive, with an average of 3.5 on a scale from 1 (worst) to 5 (best).

**Table 3:** Subjective quality of the Easypod AR mobile app.

Score	Description	Respondents, n (%)
5	Definitely, I would recommend this app to everyone.	11 (48)
4	There are many people I would recommend this app to.	5 (22)
3	Maybe, there are several people whom I would recommend it to.	5 (22)
2	There are very few people I would recommend this app to.	2 (8)
1	Not at all, I would not recommend this app to anyone.	0 (0)

### Subjective Feedback and COVID-19

Fourteen of 23 participants reported that they “especially liked” or “strongly liked” the potential to reduce fear of injections (eg, “Relieving anxiety about injections”). Also, one nurse reported that the app might be useful for training children and parents together (eg, “Easy to use, parents and children can engage training together”).

As explained in the Methods, we included some questions about the impact of COVID-19, because the introduction of Easypod AR occurred at the same time as the global pandemic. Indeed, of the participating nurses, 12 answered that yes, they believed such a mobile solution could improve patient education in the context of such a public health situation.

## Discussion

### Principal Findings

This study investigated the experiences of nurses who used a mobile app to support patient training and education within a PSP that was mostly virtual (phone-based). The results are quite encouraging in terms of the positive feedback received from the nurses; these results are aligned with previous studies on the use of AR in nursing care [21] and pediatric patient education [18-20]. Furthermore, the average rating of the app was quite positive when compared with examples used in MARS testing [22]. The results also showed areas for improvement in terms of technical aspects that might also be related to the heterogeneity of setups (eg, different smartphone capabilities and internet connections). Qualitative feedback suggested adding gamification elements, which have the potential to support adherence to r-hGH therapy [27]. Our study adds to the previous literature due to its international nature, with nurses participating from different countries who all worked in specialized care (pediatric endocrinology) using telehealth. Importantly, this shows the potential of AR-based approaches to support patient education across different health care systems.

International recommendations state that nursing involvement is needed in the development and application of artificial intelligence–based technologies in nursing [28]. However, research indicates that nurses are traditionally not sufficiently involved in the development of these technologies [29]. This study also shows the potential of involving nurses in the evaluation and assessment of digital health solutions that can, in fact, help increase digital health literacy [30] among those HCPs who are involved in patient education and are consequently also able to advise patients on mobile health solutions. This is especially important in growth-related disorders, where there have been concerns about the quality of some available mobile apps [12].

### Limitations

Our study has some limitations. The use of a survey (MARS) has many inherent limitations (eg, a lack of detailed qualitative feedback). Also, here, a modified version of the survey was used; in particular, we did not address the *information* dimension in the standard MARS survey, as it was not pertinent to the research objective, and we added a few complementary questions (eg, impact of the COVID-19 pandemic). Furthermore, our study did not address the objective impact on patient education outcomes (eg, reducing errors or promoting adherence), as this would have required a separate study design. Therefore, future research is needed to explore the patient’s perspective.

Our study addresses the acceptability of an AR-based mobile solution to support nurse-led patient education from the nurses’ perspective, but we did not address the quantifiable impact in nursing practice. Although there is evidence supporting the use of AR in nursing [21], few studies have looked into how this mobile solution impacts clinical encounters. An example outside of the domain of pediatric endocrinology is Sisom (from the Norwegian phrase “Si det Som det er,” meaning “Tell it how it is”), a computer-based animated tool that analyzed the impact of a mobile solution in communication between pediatric oncology patients and nurses [31,32]. Future research using an experimental study design could investigate how mobile solutions impact the quality of nursing encounters in patient education for r-hGH therapy.

### Conclusions

The Easypod AR mobile app was well-received by nurses involved in the training of patients with GHD for the administration of r-hGH, especially with regard to nurses’ perceptions about the potential to improve patient education, improve self-management, relieve anxiety about injections, and encourage patients and their families to seek further support from PSPs.

## Appendix

Multimedia Appendix 1 Adapted MARS survey.

Multimedia Appendix 2 Survey data.

## Abbreviations

AR: augmented reality
GH: growth hormone
GHD: growth hormone deficiency
HCP: health care professional
MARS: Mobile App Rating Scale
PSP: patient support program
r-hGH: recombinant human growth hormone

## Data Availability

All data generated or analyzed during this study are included in this published article or the Multimedia Appendices 1 and 2.
