# Supplementary material for: An Augmented Reality Mobile App (Easypod AR) as a Complementary Tool in the Nurse-Led Integrated Support of Patients Receiving Recombinant Human Growth Hormone: Usability and Validation Study
Source: JMIR Nurs. 2023 Apr 21;6:e44355. doi: 10.2196/44355 (PMC10163401; doi:10.2196/44355)
Supplement: Multimedia Appendix 1 [file nursing_v6i1e44355_app1.pdf]

Supplementary File 1: Adapted MARS survey

Evaluation of easypod® AR (Augmented Reality)

Your collaboration is essential to the realisation of this project and the research team would like to thank you for it. Before giving your informed consent, it is important that you understand why the research is being done, what it involves, and what your rights and obligations are.

INFORMATION ABOUT THE STUDY

Your participation in the present study conducted online at the University of Valencia (Spain) in collaboration with Merck Group is totally voluntary. We ask you to provide us your agreement to participate in the study by filling out the present consent form. You are free to withdraw anytime and without any justification and/or prejudices. Please, take a close look at the following paragraphs and do not hesitate to ask us if you have any questions or doubts.

What is the objective of the present study?
You are invited to participate in a research study aiming to understand better what makes the mobile solution easypod® AR useful for training of patients that are supported by Saizen Patient Support Programmes®. easypod® AR (Augmented Reality) is an App with an interactive digitized version of the easypod® 's (device) Instructions For Use. It provides instructions and details on how to correctly administer treatment using the easypod® device. Check it on this website:
https://play.google.com/store/apps/details?id=de.merck.easypodarapp.googleplay&hl=en&gl=US

The study consists of an anonymized web questionnaire where you will be asked to provide your insights on the utility and usability of easypod® AR. This research is very important since increasingly nurses are provided with digital health tools to complement their patient education work, and there is a need to better capture the experience of nurses in such type of digital health applications.

Are there any associated risks?
Following the current knowledge, the assessment of this study does not imply any risk for the participant.

Is the study voluntary? Can I withdrawal from the project?
After reading and understanding the information contained in the project description, your signature on this form indicates that you agree to participate in the project, without any external constraints or pressure. Participation in this study is voluntary. Your decision whether or not to participate will not affect your current or future relations with Merck AkG. If you decide to participate, you are free to withdraw at any time without affecting those relationships. You will not have to provide any justification for your withdrawal, and you will not incur any liability.

How is data managed?
This questionnaire is anonymous and consequently, we will not be able to track it to you. In case,

you provide in the free text forms of the questionnaire personal information (i.e. names, emails) that will be removed from the research database.

The researchers that are responsible for the present study are:
1) Professor Rosa María Baños Rivera, Department of Personality, Psychological Assessment and Treatment, Universitat de Valencia (Valencia), Tel.; +34 963 864 412, e-mail: banos@uv.es.
2) Dr. Ekaterina Koledova, Merck AKG, ekaterina.koledova@merckgroup.com

1. Are you a nurse working in a Saizen ® Patient Support Program where the app easypod® AR has been used? *

- ◯ Yes
- ◯ No

2. Do you agree to participate in the study?

   1) I acknowledge that I have been informed about the purpose and modalities of the project, as well as the advantages, risks and disadvantages of the project.
   2) I acknowledge having obtained the answers to my questions in a clear and precise manner and I have the opportunity to ask additional questions about the project or my rights at any time during the project by contacting the contact persons.
   3) I acknowledge that I have been informed that I can terminate my participation in the project at any time without any reason to give, by simply contacting the contacts listed above. *

- ◯ Yes
- ◯ [illegible]

3. We are sorry but you are not eligible for the study, you need to consent and being a nurse involved in a Saizen® PSP. If you have any feedback, feel free to add a text response below.

4. Where have you been working with Saizen® PSP? *

- ◯ Hong Kong
- ◯ Taiwan
- ◯ Germany
- ◯ Singapore
- ◯ UK
- ◯ Australia
- ◯ South Korea

Engagement

fun, interesting, customisable, interactive (e.g. sends alerts, messages, reminders, feedback, enables sharing), well-targeted to audience

5. Entertainment: Is the easypod® AR fun/entertaining to use? Does it use any strategies to increase
   engagement through entertainment (e.g. through gamification)

- ◯ 1 Dull, not fun or entertaining at all
- ◯ 2 Mostly boring
- ◯ 3 OK, fun enough to entertain user for a brief time (< 5 minutes)
- ◯ 4 Moderately fun and entertaining, would entertain user for some time (5-10 minutes total)
- ◯ 5 Highly entertaining and fun, would stimulate repeat use

6. Interest: Is the app interesting to use? Does it use any strategies to increase engagement by
   presenting its content in an interesting way?

- ◯ 1 Not interesting at all
- ◯ 2 Mostly uninteresting
- ◯ 3 OK, neither interesting nor uninteresting; would engage user for a brief time (< 5 minutes)
- ◯ 4 Moderately interesting; would engage user for some time (5-10 minutes total)
- ◯ 5 Very interesting, would engage user in repeat use

7. Customisation: Does it provide/retain all necessary settings/preferences for apps features (e.g.
sound, content, notifications, etc.)?

- ◯ 1 Does not allow any customisation or requires setting to be input every time
- ◯ 2 Allows insufficient customisation limiting functions
- ◯ 3 Allows basic customisation to function adequately
- ◯ 4 Allows numerous options for customisation
- ◯ 5 Allows complete tailoring to the individual's characteristics/preferences, retains all settings

8. Interactivity: Does it allow user input, provide feedback, contain prompts (reminders, sharing
options, notifications, etc.)? Note: these functions need to be customisable and not
overwhelming in order to be perfect.

- ◯ 1 No interactive features and/or no response to user interaction
- ◯ 2 Insufficient interactivity, or feedback, or user input options, limiting functions
- ◯ 3 Basic interactive features to function adequately
- ◯ 4 Offers a variety of interactive features/feedback/user input options
- ◯ 5 [illegible]

9. Target group: Is the app content (visual information, language, design) appropriate for your
target audience?

- ◯ 1 Completely inappropriate/unclear/confusing
- ◯ 2 Mostly inappropriate/unclear/confusing
- ◯ 3 Acceptable but not targeted. May be inappropriate/unclear/confusing
- ◯ 4 Well-targeted, with negligible issues
- ◯ 5 Perfectly targeted, no issues found

Functionality

app functioning, easy to learn, navigation, flow logic,
and gestural design of app

10. Performance: How accurately/fast do easypod® AR features (functions) and components
(buttons/menus) work?

- ◯ 1. App is broken; no/insufficient/inaccurate response (e.g. crashes/bugs/broken features, etc.)
- ◯ 2. Some functions work, but lagging or contains major technical problems
- ◯ 3. App works overall. Some technical problems need fixing/Slow at times
- ◯ 4. Mostly functional with minor/negligible problems
- ◯ 5. Perfect/timely response; no technical bugs found/contains a 'loading time left' indicator

11. Ease of use: How easy is it to learn how to use easypod® AR; how clear are the menu labels/icons and
instructions?

- ◯ 1. No/limited instructions; menu labels/icons are confusing; complicated
- ◯ 2. Useable after a lot of time/effort
- ◯ 3. Useable after some time/effort
- ◯ 4. Easy to learn how to use the app (or has clear instructions)
- ◯ 5. Able to use app immediately; intuitive; simple

12. Navigation: Is moving between screens logical/accurate/appropriate/ uninterrupted; are all necessary screen links present?

- ◯ 1. Different sections within the app seem logically disconnected and random/confusing/navigation is difficult
- ◯ 2. Usable after a lot of time/effort
- ◯ 3. Usable after some time/effort
- ◯ 4. Easy to use or missing a negligible link
- ◯ 5. Perfectly logical, easy, clear and intuitive screen flow throughout, or offers shortcuts

13. Gestural design: Are interactions (taps/swipes/pinches/scrolls) consistent and intuitive across all components/screens?

- ◯ 1. Completely inconsistent/confusing
- ◯ 2. Often inconsistent/confusing
- ◯ 3. OK with some inconsistencies/confusing elements
- ◯ 4. Mostly consistent/intuitive with negligible problems
- ◯ 5. Perfectly consistent and intuitive

Aesthetics

graphic design, overall visual appeal, colour scheme, and stylistic consistency

14. Layout: Is arrangement and size of buttons/icons/menus/content on the screen appropriate or zoomable if needed? *

- ◯ 1. Very bad design, cluttered, some options impossible to select/locate/see/read device display
- ◯ 2. Bad design, random, unclear, some options difficult to select/locate/see/read
- ◯ 3. Satisfactory, few problems with selecting/locating/seeing/reading items or with minor screensize problems
- ◯ 4. Mostly clear, able to select/locate/see/read items
- ◯ 5. Professional, simple, clear, orderly, logically organised, device display optimised. Every design

15. Graphics: How high is the quality/resolution of graphics used for buttons/icons/menus/content? *

- ◯ 1. Graphics appear amateur, very poor visual design - disproportionate, completely stylistically
- ◯ 2. Low quality/low resolution graphics; low quality visual design – disproportionate, stylistically
- ◯ 3. Moderate quality graphics and visual design (generally consistent in style)
- ◯ 4. High quality/resolution graphics and visual design – mostly proportionate, stylistically consistent

16. Visual appeal: How good does the app look? *

- ◯ 1. No visual appeal, unpleasant to look at, poorly designed, clashing/mismatched colours
- ◯ 2. Little visual appeal – poorly designed, bad use of colour, visually boring
- ◯ 3. Some visual appeal – average, neither pleasant, nor unpleasant
- ◯ 4. High level of visual appeal – seamless graphics – consistent and professionally designed
- ◯ 5. As above + very attractive, memorable, stands out; use of colour enhances app features/menus

Impact of easypod® AR

to assess the perceived impact of the app on the user's knowledge, attitudes, intentions to change as well as the likelihood of actual change in the target health behaviour

17. Question about impact of the app on the user's knowledge, attitudes, intentions to change as well as the likelihood of actual change in the target health behaviour *

	Strongly disagree	Disagree	Neutral	Agree	Strongly agree
Awareness: The use of easypod® AR app is likely to increase awareness of the importance of addressing the treatment procedure properly and safely (f.e. attach the needle, correct dose, rotation of injection site, injection delivery with waiting time on the skin, detaching the needle, care after injection)	○	○	○	○	○
Knowledge: The use of easypod® AR app is likely to increase knowledge/understanding of having the correct treatment procedure properly and	○	○	○	○	○

Strongly disagree Disagree Neutral Agree Strongly agree

safely.					
Attitudes: The use of easypod® AR is likely to change attitudes toward using the device properly and in a safe way	○	○	○	○	○
Intention to change: The use of easypod® AR is likely to increase intentions/motivation to follow the correct treatment procedure	○	○	○	○	○
Help seeking: The use of easypod® AR is likely to encourage further help-seeking for correct treatment procedure the Patient Support Programme (PSP)	○	○	○	○	○
Behaviour change: Use of easypod® AR is likely to increase the proper and safe administration of this treatment procedure	○	○	○	○	○
Emotions: the use of [illegible]	Strongly disagree	Disagree	Neutral	Agree	Strongly agree

easypod® AR is likely to decreases the fear and anxiety of using this treatment procedure	◯	◯	◯	◯	◯

App subjective quality

18. Would you recommend this app to people who might benefit from it?

- ◯ 1. Not at all / I would not recommend this app to anyone
- ◯ 2. - There are very few people I would recommend this app to
- ◯ 3. Maybe / There are several people whom I would recommend it to
- ◯ 4. - There are many people I would recommend this app to
- ◯ 5. Definitely / I would recommend this app to everyone

19. What is your overall star rating of the app?

One of the worst apps I've us ed ☆ ☆ ☆ ☆ ☆ One of the best apps I've us ed

20. Has the use of easypod® AR been more relevant during the recent COVID-19 crisis-related restrictions?

- ◯ Yes
- ◯ Maybe
- ◯ No

21. Has COVID19 crisis changed significantly your onboarding of patients and training activities on the easypod® device?

- ◯ Yes
- ◯ Maybe
- ◯ No

22. Do you believe that having easypod® AR has allowed you to train new patients remotely overcoming COVID19 related restrictions?

- ◯ Yes
- ◯ Maybe
- ◯ No

23. After COVID19 do you believe that easypod® AR allows you to be more efficient on the onboarding of new patients remotely?

- ◯ Yes
- ◯ Maybe
- ◯ No

24. Please enter any positive feedback about your experience with easypod® AR

25. Please enter any feedback suggestion for the improvement of easypod® AR

This content is neither created nor endorsed by Microsoft. The data you submit will be sent to the form owner.

Microsoft Forms
